# Time Invariant Surface Roughness Evolution during Atmospheric Pressure Thin Film Depositions

**DOI:** 10.1038/srep19888

**Published:** 2016-01-27

**Authors:** Thomas Merkh, Robert Spivey, Toh Ming Lu

**Affiliations:** 1 Rensselaer Polytechnic Institute, Department of Physics, Applied Physics and Astronomy, and Center for Materials, Devices, and Integrated Systems, 110 8th St, Troy NY 12180, United States

## Abstract

The evolution of thin film morphology during atmospheric pressure deposition has been studied utilizing Monte Carlo methods. Time invariant root-mean-squared roughness and local roughness morphology were both observed when employing a novel simulation parameter, modeling the effect of the experimental high pressure condition. This growth regime, where the surface roughness remains invariant after reaching a critical value, has not been classified by any existing universality class. An *anti-shadowing* growth mechanism responsible for this regime occurs when particles undergo binary collisions beneath the surface apexes. Hence, this mechanism is applicable when the mean free path of the depositing species is comparable to the amplitude of the surface features. Computationally this has been modeled by allowing particles to change direction at a specified height above the local film surface. This modification of the incoming flux trajectory consequently has a dramatic smoothening effect, and the resulting surfaces appear in agreement with recent experimental observations.

The morphological evolution of growing material layers is of continuous interest in the scientific community both from theoretical and practical point of views^1^,^2^,^3^,^4^,^5^. The interest thrives due to the substantial effect surface morphology has on the optical, electrical, and mechanical properties of a film. Naturally, many methods have been developed to assist characterizing and predicting morphologies. Among these, scaling relationships involving measurable surface features, such as the root-mean-squared roughness *ω*, hold strong predictive power without concerning the detailed properties of the material in question. These relationships give rise to scaling exponents, which may only posses a specific set of values that depend on the dimensionality and symmetry of the growth dynamics. Furthermore, these exponents allow for seemingly unrelated growth phenomenon to be categorized within the same universality class. Growth within a universality class is predictable and admits reliable surface fabrication^1^,^2^,^3^,^6^.

Of particular interest is the roughness exponent, *α*, and the growth exponent *β*. The latter arises from the relation, *ω*(*t*) ~ *t*^*β*^, where *ω*(*t*) = 〈[*h*(*r*′, *t*) − 〈*h*(*t*)〉]^2^〉 and *h*(*r*, *t*) is the surface height at position *r* at time *t*. The former describes the local roughening behavior, and may be defined by first considering the height-height correlation function (HHCF),


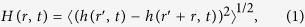


where 〈···〉 denotes a statistical average. For self-affine surface growth problems, this function behaves like


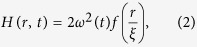


where


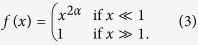


Here, *ξ* denotes the maximum horizontal length at which surface heights are correlated, given by the scaling relation *ξ* ~ *t*^*β*/*α*^ 2,3. The meaning of *α* is not as elusive as the mathematics appear; *α* describes the frequency of local height fluctuation, with a value near unity corresponding to a slow oscillatory surface and a value near zero corresponding to a rapid oscillatory surface^6^,^7^. Knowing the experimental conditions under which these exponents maintain a certain value allows one to fabricate films with predictable morphological properties.

A recent analysis of organosilicone films grown using plasma enhanced chemical vapor deposition (CVD) in ambient pressure shows the produced surfaces possess unique scaling characteristics unlike any previous universal classes^8^,^9^. Namely, the isotropic surfaces displayed time independent surface roughness characterized by *β* ≈ 0 and constant *α*. For a full description of the deposition, refer to references^8^,^10^,^11^. One critical aspect of this work was the high pressure environment in which depositions were performed. High pressure conditions have not been included in previous theoretical work predicting surface roughness as a function of experimental parameters. In the subsequent discussion, Monte Carlo methods are employed in exploring a possible growth mechanism that can produce the time invariant roughness seen during high pressure deposition. The following proposed mechanism is able to explain the salient features during atmospheric pressure deposition.

## Methods

### Monte Carlo Model

**Figure 1:**
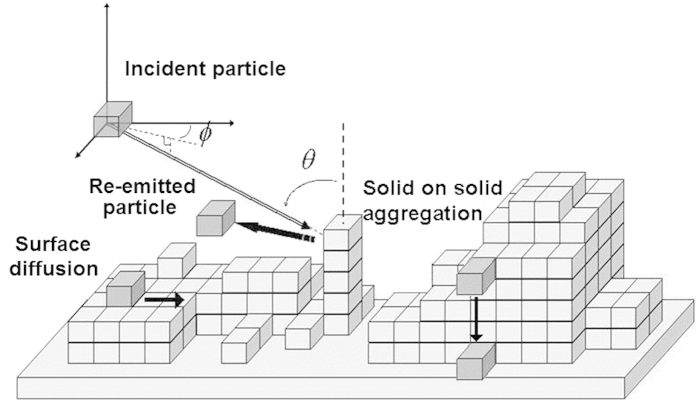
Monte Carlo growth scheme showing several simulation processes. Solid on solid aggregation can be seen as a side sticking particle sides downward until reaching the surface. This side sticking would be allowed during ballistic deposition. Additionally, surface diffusion can be seen as a particle hops across the surface reaching a more stable state.

A full three dimensional Monte Carlo simulation of thin film growth was employed to simulate film deposition under high pressure deposition conditions. A 512 by 512 lattice with periodic boundary conditions was used, and each particle deposited at position (*x*, *y*) incremented the surface height profile *h*(*x*, *y*; *t*) by unity. Two general aggregation schemes were investigated. The first was the solid on solid (SOS) model in which the surface height profile was strictly single valued. In other words, instances of overhanging particles were disallowed as all side-sticking particles were required to slide downward in place until they lay on top of the surface. Second, ballistic aggregation was investigated, where instances of overhangs were allowed. The total deposition process consisted of 10^8^ deposition events equally distributed over 1,500 time steps where each particle began at a random (*x*, *y*) location and one lattice unit above the maximum surface height, then propagated toward the substrate in a straight line. Note that this particular deposition rule was developed for situations where the mean free path of the depositing particle is much greater than the magnitude of the surface features. The trajectory of the impending particles was determined according to a predefined probability distribution. For sputter deposition and low pressure CVD, the flux distribution is generally assumed to be of a cosine form,


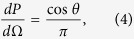


where Ω is the solid angle and *θ* is the trajectory direction with respect to the substrate normal. Random *θ* values were generated by 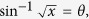 where *x* was a uniformly distributed random number between 0 and 1^12^,^13^. After impacting the surface, a particle had a probabilistic chance to deposit, determined by the sticking coefficient *s*_*o*_ between 0 and 1. These simulations used *s*_*o*_ = 1, meaning particles deposited on impact. Previous investigations have included higher order sticking coefficients accounting for secondary impact events. After a particle was deposited on the surface, a number of particles within the nearby vicinity were chosen to diffuse. Each selected particle was then assigned a random energy between 0 and 1. If this energy was less than 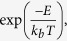 where *E* = *E*_*o*_ + *cE*_*n*_, the particle had sufficient energy to diffuse to a nearby location^3^,^14^,^15^,^16^. Here we used *E*_*o*_ = 0.08 eV as the activation energy for diffusion, *E*_*n*_ = 0.05 eV as the bond energy between neighboring sites, *T* = 300 K for the temperature, and *c* is the integer number of occupied sites neighboring the diffusive particle. To illustrate this process, suppose the particle chosen for diffusion has one neighboring particle. Then the particle’s randomly assigned energy between 0 and 1 must be less than 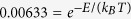, where *E* = 0.08 + 1 × (0.05), for diffusion to occur. The number of possible diffusion events after each deposition event is dependent upon the material in question. For silicon depositions at standard temperature, 100 diffusion events had been used previously to model the strength of surface diffusion^17^. These simulations had taken the same value. If one uses the rate in the example to determine the likelihood of a particle diffusing after 100 attempts, the probability is around a half. We did not intend for diffusion to be a strong presence in these simulations, as we believe that diffusion is not the dominant smoothening process. Rather, we observe that when the average collision height of the incoming particles is below the height of the surface features, a time invariant surface roughness occurs. Figure 1 provides a visualization of several simulation processes. For further description of the simulation, refer to references^15^,^18^,^19^,^20^,^21^.

### High Pressure Modification

**Figure 2:**
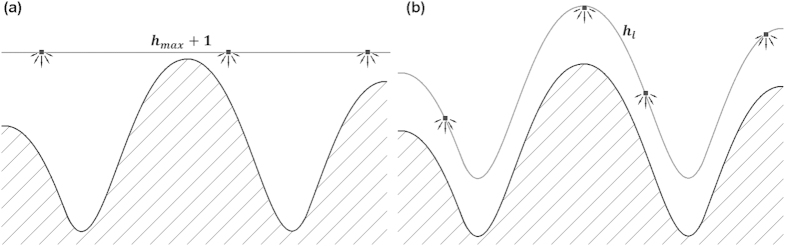
(**a**) The conventional growth process. Depositing particles begin their trajectory starting strictly at *h*_*max*_ + 1, one lattice unit above the maximum surface height. Initializing particles an additional height *h*_*a*_ above *h*_*max*_ has no effect on morphology because a lateral translation by *h*_*a*_tan(*θ*) results in the same flux at the surface, where *θ* is the trajectory angle. Because the initial (*x*, *y*) position of the particle is chosen randomly, arbitrary translations are permitted. (**b**) The modified growth process assumes a mean collision height above the surface at height *h*_*l*_, where the particles begin their trajectory toward the surface.

The conventional Monte Carlo method described above was inadequate in reproducing the observed growth under high pressure. No combination of the parameters commonly thought to counteract roughening, such as a low sticking coefficient^22^, strong surface diffusion^23^,^24^,^25^,^26^,^27^, or altered flux distribution^28^,^29^, was capable of producing time invariant surface roughness. Low sticking coefficients initially display *β* ≈ 0, however after a sufficient number of particles have deposited, the surface displays roughening and non-invariant growth. The modification necessary was found to be related to the depositing particle’s mean free path. Collisions between gas molecules and impending particles effectively alters the angular flux distribution at the surface, leading to a new smoothening phenomenon called *anti-shadowing*. These persistent collisions nearby the surface were modeled by initializing the particle’s starting location, and likewise its angular trajectory, at specified height above the surface profile. This specified height, denoted by *h*_*l*_, can qualitatively be recognized as the mean collision height above the surface. Figure 2 compares the conventional simulation process to the modified process. For low pressure deposition, *h*_*l*_ is much greater than the amplitude of the surface features and the conventional simulation method suffices. However in a high pressure environment, the proposed modification may be necessary to account for the reduced mean free path of the particles and the sporadically varying flux trajectories nearby the surface. The simulated surfaces in the forthcoming section have been revised with this modification, and the parameters *s*_*o*_ = 1 and cos (*θ*) flux distribution were used on a template with initial *ω* = 0.68 lattice units.

## Results

**Figure 3:**
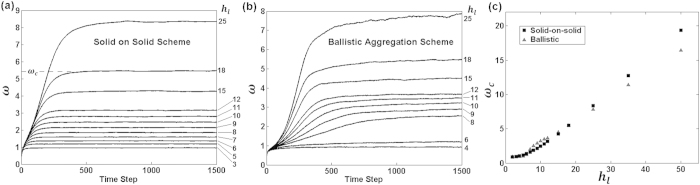
(**a**) The *ω*(*t*) function shown for each SOS simulation over 1,500 time steps. It is evident that after reaching a critical roughness, which appears to be a function of *h*_*l*_ (denoted on the right of each plot), *ω* thereafter remains invariant. (**b**) The *ω*(*t*) function shown for each ballistic simulation. The *ω*_*c*_ occurrence is independent of the aggregation scheme used. (**c**) Critical roughness values as a function of *h*_*l*_. This relationship appears linear except for *h*_*l*_ values very near the surface. These values may not be important in practice because achieving *h*_*l*_ values so close to the surface would require extreme deposition pressures.

The proposed modification was studied in terms of the effect *h*_*l*_ induces on the surface roughness. Twelve nearly identical simulations were performed and each surface’s *ω* was recorded after each time step. The only alteration between each simulation was the *h*_*l*_ parameter. Figure 3(a,b) display the time evolution of *ω* for each SOS and ballistic simulation respectively. Each simulation is labeled by its respective *h*_*l*_ value on the right. It is evident that after a critical roughness, *ω*_*c*_, was reached, the surface roughness remained invariant with time. A flattening of the *ω*(*t*) function indicates that *β* ≈ 0 according to power law *ω*(*t*) ~ *t*^*β*^. Further, it seems that *ω*_*c*_ is dependent upon the *h*_*l*_ parameter. The nature of this dependence is demonstrated in Fig. 3(c). Forgo the extremely small values of *h*_*l*_, this relationship appears linear. The result is reassuring in that the simulation parameter *h*_*l*_ is thought to be proportional to mean free path. This implies that *h*_*l*_ decreases as a function of increasing pressure, giving rise to the smooth surfaces produced by high pressure CVD. In other words, as the mean collision height above the surface shrinks, the surface reaches its critical roughness more rapidly, resulting in a smoother surface.

**Figure 4:**
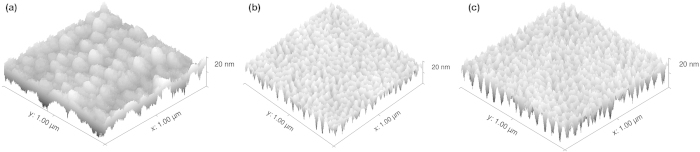
(**a**) The initial substrate used in simulation. The substrate began with *ω* = 0.68 lattice units, and *α* = 0.24. This particular example used the parameter *h*_*l*_ = 8 lattice units. (**b**) The simulated film after 1,000 time steps. The morphology shown is the statistically invariant morphology observed after *ω*_*c*_ has been reached. (**c**) The simulated film after 1,500 time steps.

**Figure 5:**
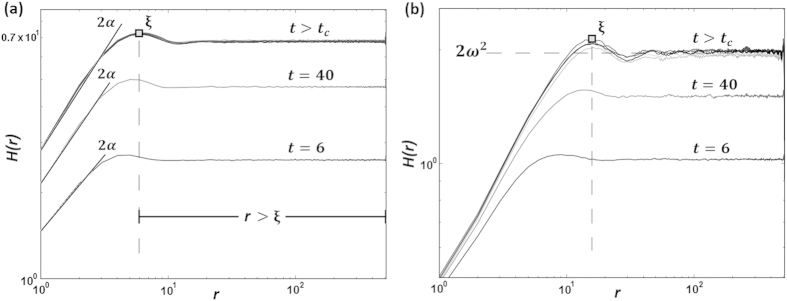
(**a**) The log scale HHCF for the SOS simulation discussed in Fig. 4. *H*(*r*; *t*) was plotted at various time steps during the simulation as indicated on the plot. After *t* > *t*_*c*_, the HHCF remains invariant as well as the slope in the region *r* < *ξ*. This slope provides twice the value of *α*. The constant slope seen after *t* > *t*_*c*_ indicates that the local roughness remains constant after the critical roughness has been reached. (**b**) The log scale HHCF for the ballistic simulation with *h*_*l*_ = 8. Similar to the SOS case, *α* remains invariant when *t* > *t*_*c*_.

Figure 4 displays the morphology of the film before, during, and after simulation. The morphology present at the end of the simulation is the same statistically invariant morphology maintained after the critical roughness has been reached. Although the morphology itself continues to evolve, the root-mean-squared roughness and the frequency of local height fluctuations both remain constant. This information is conveyed by the value of *α*. The *α* value was determined by measuring the half slope of the log scale HHCF in the region *r* < *ξ*, described by equations (1) and (2). Figure 5 displays the log scale HHCF for one simulation, plotted at various times. Before the critical roughness has been reached, the slope of *H*(*r*) evolves slightly. However, denoting the time at which the critical roughness has been reached as *t*_*c*_, for times *t* > *t*_*c*_, the slope value of *H*(*r*) remains invariant with time. This is depicted in Fig. 5, as the *H*(*r*) function lays closely on top of itself for all *t* > *t*_*c*_. These combined results show that *β* ≈ 0 and *α* remains constant after a certain *ω*_*c*_ has been reached.

## Discussion

It is believed that the growth mechanism of *anti-shadowing* occurs when the mean free path of the depositing particles is comparable to the surface features. The mean collision height above the surface, at which the incoming particles change trajectory, plays a crucial role in determining the local morphology and root-mean-squared roughness of the film. The empirical relationship linking the experimental deposition pressure to the simulation parameter *h*_*l*_ has not been established. This relationship remains inaccessible due to the complexities of the mean free path during deposition. In the case of CVD deposition, for example, the varying gas density throughout the chamber leads to a complex analysis of the mean free path^30^. Furthermore, the varying density is conjointly a function of the chamber geometry and inlet rate, making each instance of deposition unique. CVD presents additional challenges due to the intricacies of common precursor gases, such as Hexamethyldisiloxane (HMDSO) and tetraethyl orthosilicate (TEOS)^8^,^9^. However, to illustrate when this regime is expected, we can estimate the lowest pressure needed to exhibit the anti-shadowing effect for a given surface height feature. For a surface height feature in the tens of nm, as we have demonstrated in this work, atmospheric pressure (with the mean free path on the order of tens of nm) is needed to prevent roughening during growth. For a surface height feature of one micron or higher, the lowest pressure that would give a comparable mean free path is about 50 Torr for a common gas such as Ar. In other words, under this pressure, the surface with a height feature larger than one micron may cease roughening and maintain its approximate roughness as the deposition continues. We do not expect this regime to be relevant to the sputter deposition experiments because the pressure is limited to tens of mTorr.

Nevertheless, these results predict the behavior of the scaling exponents *β* and *α*, leading to a new universality class of growth. Quantitatively, this high pressure universality class is defined by *β* ≈ 0 and a constant *α*. This class exists outside of the specific diffusion parameter values, angular flux distribution, and aggregation scheme used. The crucial parameter that brings about this class of growth is the mean collision height of depositing particles. Qualitatively, it is observed that when the mean collision height is comparable to the size of the surface features, time invariant growth properties arise. Together, these results provide a microscopic explanation of the growth mechanism that gives rise to the morphologies seen during deposition in a high pressure environment.

## Additional Information

**How to cite this article**: Merkh, T. *et al*. Time Invariant Surface Roughness Evolution during Atmospheric Pressure Thin Film Depositions. *Sci. Rep.* **6**, 19888; doi: 10.1038/srep19888 (2016).

## References

[b1] Family F. & Vicsek T. Scaling of the active zone in the eden process on percolation networks and the ballistic deposition model. J. Phys. A: Math. Gen. 18 (1985).

[b2] Family F. & Vicsek T. Dynamics of Fractal Surfaces (World Scientific, Singapore, 1991). URL http://books.google.com/books?id=-rHqHnwwVyYC. (Date accessed 10/13/2015).

[b3] Barabasi A.-L. & Stanley H. Fractal Concepts in Surface Growth (Cambridge University Press, 1995).

[b4] Meakin R. Fractals, Scaling, and Growth Far from Equilibrium (Cambridge University Press, 1998). URL http://books.google.com/books?id=cWmbNYSQDKoC. (Date accessed 10/13/2015).

[b5] Zhao Y.-P., Wang G.-C. & Lu T.-M. Characterization of Amorphous and Crystalline Rough Surface: Principles and Applications (Academic Press, 2001).

[b6] Pelliccione M. & Lu T.-M. Evolution of Thin Film Morphology (Springer, 2008).

[b7] Krim J. & Indekeu J. O. Roughness exponents: A paradox resolved. Phys. Rev. E 48, 1576–1578 (1993). URL http://link.aps.org/doi/10.1103/PhysRevE.48.1576. (Date accessed 10/13/2015). 10.1103/physreve.48.1576 9960753

[b8] Premkumar P. A. . Surface dynamics of sio2-like films on polymers grown by dbd assisted cvd at atmospheric pressure. Plas. Proc. and Poly. 9, 1194–1207 (2012). URL http://onlinelibrary.wiley.com/doi/10.1002/ppap.201200016/pdf. (Date accessed 10/13/2015).

[b9] Premkumar P. A. . Morphological description of ultra-smooth organo-silicone layers synthesized using atmospheric pressure dielectric barrier discharge assisted pecvd. Plas. Proc. and Poly. 10, 313–319 (2013). URL http://onlinelibrary.wiley.com/doi/10.1002/ppap.201200101/pdf. (Date accessed 10/13/2015).

[b10] Starostine S., Aldea E., Vries H., Creatore M. & Sanden M. Atmospheric pressure barrier discharge deposition of silica-like films on polymeric substrates. Plas. Proc. and Poly. 4, 440–444 (2007).

[b11] Starostin S. . High current diffuse dielectric barrier discharge in atmospheric pressure air for the deposition of thin silica-like films. Appl. Phy. Let. 96 (2010). URL http://dx.doi.org/10.1063/1.3310024. (Date accessed 10/13/2015).

[b12] Suetsugu Y. Application of the monte carlo method to pressure calculation. J. of Vac. Sci. and Tech. A 14, 245–250 (1996). URL http://scitation.aip.org/content/avs/journal/jvsta/14/1/10.1116/1.579927. (Date accessed 10/13/2015).

[b13] Greenwood J. The correct and incorrect generation of a cosine distribution of scattered particles for monte-carlo modelling of vacuum systems. Vacuum 67, 217–222 (2002). URL http://www.sciencedirect.com/science/article/pii/S0042207×02001732. (Date accessed 10/13/2015).

[b14] Das Sarma S. & Tamborenea P. A new universality class for kinetic growth: One-dimensional molecular-beam epitaxy. Phys. Rev. Lett. 66, 325–328 (1991). URL http://link.aps.org/doi/10.1103/PhysRevLett.66.325. (Date accessed 10/13/2015). 10043777 10.1103/PhysRevLett.66.325

[b15] Karabacak T., Zhao Y.-P., Wang G.-C. & Lu T.-M. Growth-front roughening in amorphous silicon films by sputtering. Phys. Rev. B 64, 085323 (2001). URL http://link.aps.org/doi/10.1103/PhysRevB.64.085323. (Date accessed 10/13/2015).

[b16] Amar J. G., Family F. & Lam P.-M. Dynamic scaling of the island-size distribution and percolation in a model of submonolayer molecular-beam epitaxy. Phys. Rev. B 50, 8781–8797 (1994). URL http://link.aps.org/doi/10.1103/PhysRevB.50.8781. (Date accessed 10/13/2015). 10.1103/physrevb.50.8781 9974899

[b17] Ye D., Karabacak T., Picu R., Wang G. & Lu T.-M. Uniform si nanostructures grown by oblique angle deposition with substrate swing rotation. Nanotechnology 16, 1717 (2005).

[b18] Pelliccione M., Karabacak T., Gaire C., Wang G.-C. & Lu T.-M. Mound formation in surface growth under shadowing. Phys. Rev. B 74, 125420 (2006). URL http://link.aps.org/doi/10.1103/PhysRevB.74.125420. (Date accessed 10/13/2015).

[b19] Karabacak T., Zhao Y.-P., Wang G.-C. & Lu T.-M. Growth front roughening in silicon nitride films by plasma-enhanced chemical vapor deposition. Phys. Rev. B 66, 075329 (2002). URL http://link.aps.org/doi/10.1103/PhysRevB.66.075329. (Date accessed 10/13/2015).

[b20] Karabacak T., Singh J. P., Zhao Y.-P., Wang G.-C. & Lu T.-M. Scaling during shadowing growth of isolated nanocolumns. Phys. Rev. B 68, 125408 (2003). URL http://link.aps.org/doi/10.1103/PhysRevB.68.125408. (Date accessed 10/13/2015).

[b21] Tanto B., Doiron C. F. & Lu T.-M. Large artificial anisotropic growth rate in on-lattice simulation of obliquely deposited nanostructures. Phys. Rev. E 83, 016703 (2011). URL http://link.aps.org/doi/10.1103/PhysRevE.83.016703. (Date accessed 10/13/2015). 10.1103/PhysRevE.83.016703 21405791

[b22] Zhao Y.-P., Drotar J. T., Wang G.-C. & Lu T.-M. Morphology transition during low-pressure chemical vapor deposition. Phys. Rev. Lett. 87, 136102 (2001). URL http://link.aps.org/doi/10.1103/PhysRevLett.87.136102. (Date accessed 10/13/2015). 11580608 10.1103/PhysRevLett.87.136102

[b23] Mullins W. W. Theory of thermal grooving. J. of Applied Physics 28, 333–339 (1957). URL http://scitation.aip.org/content/aip/journal/jap/28/3/10.1063/1.1722742. (Date accessed 10/13/2015).

[b24] Wolf D. E. & Villain J. Growth with surface diffusion. Europhys. Lett. 13, 389 (1990).

[b25] Sarma S. D. & Tamborenea P. A new universality class for kinetic growth: One-dimensional molecular-beam epitaxy. Phys. Rev. Lett. 66, 325–328 (1991). URL http://link.aps.org/doi/10.1103/PhysRevLett.66.325. (Date accessed 10/13/2015). 10043777 10.1103/PhysRevLett.66.325

[b26] Amar J. G., Lam P.-M. & Family F. Groove instabilities in surface growth with diffusion. Phys. Rev. E 47, 3242–3245 (1993). URL http://link.aps.org/doi/10.1103/PhysRevE.47.3242. (Date accessed 10/13/2015). 10.1103/physreve.47.3242 9960376

[b27] Yang H.-N., Zhao Y.-P., Wang G.-C. & Lu T.-M. Noise-induced roughening evolution of amorphous si films grown by thermal evaporation. Phys. Rev. Lett. 76, 3774–3777 (1996). URL http://link.aps.org/doi/10.1103/PhysRevLett.76.3774. (Date accessed 10/13/2015). 10061106 10.1103/PhysRevLett.76.3774

[b28] Hass D. D., Yang Y. Y. & Wadley H. N. G. Pore evolution during high pressure atomic vapor deposition. J. of Por. Mat. 17, 27–38 (2010).

[b29] Tan X., Ouyang G. & Yang G. W. Surface smoothing of amorphous silicon thin films: Kinetic monte carlo simulations. Phys. Rev. B 73, 195322 (2006). URL http://link.aps.org/doi/10.1103/PhysRevB.73.195322. (Date accessed 10/13/2015).

[b30] Rossnagel S. M. Gas density reduction effects in magnetrons. Journal of Vacuum Science and Technology A 6, 19–24 (1988). URL http://scitation.aip.org/content/avs/journal/jvsta/6/1/10.1116/1.574988. (Date accessed 10/13/2015).

